# *Mycobacterium tuberculosis* DosR Regulon Gene *Rv2004c* Encodes a Novel Antigen with Pro-inflammatory Functions and Potential Diagnostic Application for Detection of Latent Tuberculosis

**DOI:** 10.3389/fimmu.2017.00712

**Published:** 2017-06-24

**Authors:** Sankara Narayana Doddam, Vidyullatha Peddireddy, Niyaz Ahmed

**Affiliations:** ^1^Pathogen Biology Laboratory, Department of Biotechnology and Bioinformatics, University of Hyderabad, Hyderabad, India; ^2^Laboratory Sciences and Services Division, International Centre for Diarrhoeal Disease Research Bangladesh (icddr,b), Dhaka, Bangladesh

**Keywords:** *Mycobacterium tuberculosis*, Rv2004c, latency, DosR regulon, granuloma, cytokines, toll-like receptors

## Abstract

Approximately 1.7 billion people in the world harbor latent *Mycobacterium tuberculosis* (Mtb) with a substantial risk of progression to clinical outcome. Containment of these seed beds of Mtb is essential to eliminate tuberculosis completely in high burden settings such as India. Hence, there is an urgent need for the identification of new serological markers for detection or vaccine candidates to prevent latent tuberculosis infection (LTBI). DosR regulon antigens of Mtb might serve as attractive targets for LTBI diagnosis or vaccine development as they are specifically expressed and are upregulated during latent phase. In this study, we investigated the role of *Rv2004c*, a member of DosR regulon (exclusive to Mtb complex), in host–pathogen interaction and its immunogenic potential in LTBI, active TB, and healthy control cohorts. Rv2004c elicited strong antibody response in individuals with LTBI compared to active TB patients and healthy cohorts. Recombinant Rv2004c induced pro-inflammatory cytokine response in human peripheral blood mononuclear cells and THP-1 cells *via* NF-κB phosphorylation. Interaction of Rv2004c with toll-like receptor (TLR)-2 was confirmed using HEK-Blue hTLR-2 and pull-down assays. Rv2004c enhanced the surface expression of TLR-2 at mRNA and protein levels in THP-1 cells. Our findings revealed that Rv2004c induces strong humoral and cell mediated immune responses. Given these observations, we propose Rv2004c to be a potential diagnostic marker or an attractive vaccine candidate that can be useful against LTBI.

## Introduction

Initiation of Directly Observed Treatment Short course (DOTS) throughout India in 1997 to eliminate active tuberculosis (TB) led to the containment of TB burden to a certain extent (1, 2) but failed to eliminate/treat latent tuberculosis infection (LTBI). However, the overall prevalence of active TB is still at an alarming state in the Indian subcontinent (3). A key challenge for better control of TB primarily includes the reservoirs of LTBI- and human immunodeficiency virus (HIV)-associated TB. HIV infection usually promotes the resuscitation of LTBI. According to a recent estimate, approximately 1.7 billion population was infected with LTBI (4), which serves as the seedbed of *Mycobacterium tuberculosis* (Mtb), with a possibility of reactivating into future active TB.

Virtually all active TB cases are preceded by a period of subclinical asymptomatic infection. Hence, there is an urgent need for the screening of high-risk persons who can progress to active TB and eradication of such subclinical infections will reduce the TB transmission in high burden and low resource settings such as India and Africa (5). The high-risk groups include HIV-infected individuals, house hold contacts of active TB cases, recipients of TNF-α blockers, individuals undergoing immune suppressive therapy, diabetic patients, and people with malnutrition (6). The tuberculin skin test (TST) is still most widely used for diagnosing LTBI in low resource countries even though it has limitations. To date, there are no affordable and accurate diagnostic tools and or vaccines to combat LTBI.

Currently available novel LTBI diagnostics are based on interferon-γ release assays (IGRAs), which measure IFN-γ released by T-cells (QFT-Gold) or IFN-γ-secreting T-cells (T-SPOT.TB) that are stimulated with Mtb-specific antigens (ESAT-6, CFP-10, and TB 7.7) (7). However, IGRAs are not recommended by World Health Organization over TST in low- and middle-income countries with high background of active TB cases, for LTBI diagnosis since they are expensive, lack specificity to differentiate LTBI from active TB and cannot be used for infants and immune compromised individuals (8). Thus, the ideal LTBI diagnostic tool should be latent specific and affordable. The DosR regulon antigens of Mtb might serve as potential targets for LTBI diagnosis, as they specifically express and upregulate during the latent phase. The antigens encoded by the DosR regulon were shown to induce strong T-cell mediated immune responses in individuals with LTBI suggesting that expression of these DosR antigens is latent specific (9). Hence, evaluating the humoral immune response of DosR regulon genes might be an interesting approach to use them as serodiagnostic markers.

During latency, Mtb exists in dormant or non-replicating persistent (NRP) state and survives in hostile environment of granuloma entailing hypoxia, low pH, and high NO intermediates (10). A granuloma is a dynamic environment made up of mononuclear inflammatory cells that provide a niche for the survival of Mtb and also limit growth of bacteria by innate immune functions. Matured granuloma is composed of a core of macrophages surrounded by layers of T cells and B cells followed by a periphery of fibroblasts (11). In this state, bacilli downsize their metabolic activities resulting in decrease in RNA and protein synthesis through upregulating a regulon of 48 genes known as DosR regulon (12, 13). The two sensor kinases, DosS and DosT (members of regulon) activate the DosR, a transcriptional factor that further controls all the 48 genes in the regulon. Even though bacilli require oxygen for their physiological growth, Mtb endures in the micro aerobic conditions for a long period of time, pointing to the role of DosR regulon in bacterial adaptation and granuloma maintenance (14).

In the granulomas, the outcome of the infection is determined by the interaction of different host and pathogen-specific factors that have been implicated in the progression of the disease (15). Pattern recognition receptors (PRRs) play an important role during Mtb infection. PRRs interact with different mycobacterial cell wall components, induce cytokine responses, and control early infection. Among PRRs, toll-like receptors (TLRs) are the key receptors that recognize a wide array of conserved ligands of Mtb. Among TLRs, TLR-2 associating either with TLR-1 or TLR-6 as a heterodimer recognizes different Mtb components (16) leading to the commencement of downstream signaling events and secretion of cytokines such as TNF-α, IL-8, IL-1β, IFN-γ, and IL-12. TNF-α plays an important role in activation and recruitment of macrophages and other immune cells to the site of infection (17). Interaction of TLRs with mycobacterium cell wall components has been reported extensively (18–20). However, such interactions between TLRs and DosR regulon proteins are not yet deciphered.

Rv2004c is one of the uncharacterized proteins of DosR regulon. Localization of Rv2004c on cell surface of Mtb was reported using transmission electron microscopy (21). Interaction of peptide sequences of Rv2004c with the surface of U937 macrophages and A549 epithelial cells was demonstrated (21). Expression of Rv2004c exclusively in Mtb complex has been reported (21). However, its interaction with surface receptors and downstream signaling mechanisms are not yet deciphered. It has also been shown that Mtb upregulates Rv2004c under *in vitro* non-replicating persistence and nitric oxide stress conditions (12, 22). Transposon site hybridization mutation studies showed that Rv2004c is essential for the survival of Mtb in murine macrophages (23). The immunogenic potential of Rv2004c in latent tuberculosis infected (LTBI) individuals of Japanese population was reported (24). But, studies about humoral immune response in LTBI individuals from high burden countries such as India are lacking.

In this study, we elucidated the role of Rv2004c in host–pathogen interaction and its immunogenic response in LTBI individuals. We demonstrated that LTBI individuals exhibited higher immunoreactivity to Rv2004c when compared to individuals with pulmonary tuberculosis (PTB) and healthy controls (HCs) in urban population of Hyderabad, India. To the best of our knowledge, this is the first report pertaining to humoral immune response of a DosR regulon antigen, Rv2004c in sera samples of LTBI individuals. Its interaction with TLR-2 surface receptor, and pro-inflammatory cytokine response in human peripheral blood mononuclear cells (PBMCs) and THP-1 cells was also deciphered. Our findings support the involvement of Rv2004c in host–pathogen interactions, and its ability to serve as a potential serodiagnostic marker for LTBI.

## Materials and Methods

### Cohort Characteristics and Blood Samples

The human cohort consisted of 129 individuals (healthy = 37; latent = 52; active TB = 40) of both sexes (males = 69 and females = 60), recruited from Government Chest Hospital and Bhagwan Mahavir Hospital and Research Centre, Hyderabad. For experiments involving human samples, World Medical Association (Declaration of Helsinki) ethics code was followed. Written informed consent was collected from all the individuals, and the study was approved by both Biosafety and Ethics Committees of the University of Hyderabad and Bhagwan Mahavir Hospital and Research Centre. Individuals below 18 years, pregnant women, individuals with diabetes or any autoimmune diseases, and immunecompromised individuals were excluded from the study. Five milliliters of blood was collected from each subject, and the sera obtained were stored at −80°C. Active TB was determined by acid fast staining of the sputum samples, chest X-ray, and culture. Sampling from active TB cases was performed prior to treatment with anti-TB drugs. For LTBI testing, hospital staff from Government Chest Hospital and Bhagwan Mahavir Hospital as well as house hold contacts of active TB cases (individuals who do not have any signs and symptoms of active TB upon examination by an expert clinical physician, chest X-ray, and tested negative by Ziehl-Neelsen staining of sputum) were recruited. IGRA (QuantiFERON-TB Gold (QFT^®^), Cellestis, VIC, Australia) positive cases were considered as LTBI, and negative cases were considered as HCs.

### Cloning, Expression, and Purification of Rv2004c

*Rv2004c* gene comprising 1,497 bp length was amplified using Mtb H37Rv DNA and cloned into pET-28a vector at *BamHI* and *HindIII* restriction sites using the primers listed in Table S1 in Supplementary Material. The resultant recombinant construct was transformed into BL21(DE3) pLysS. Overnight culture was inoculated into 1 L of LB broth containing kanamycin 50 µg/ml and chloramphenicol 34 µg/ml and allowed to grow up to the OD of 0.3–0.5. Then, the culture was induced with 1 mM IPTG and harvested after 4 h of incubation. The pellet was resuspended and sonicated in lysis buffer (20 mM Tris, 300 mM NaCl) containing 0.3% sarkosyl and 1 mM PMSF. The lysate was centrifuged at 10,000 rpm for 30 min at 4°C. The supernatant collected was incubated with cobalt resin for 3 h at 4°C on a rotor spin. Later, the supernatant was loaded on to polypropylene column and washed thrice with wash buffer-1 (lysis buffer with 5 mM imidazole), wash buffer-2 (lysis buffer with 10 mM imidazole), and wash buffer-3 (lysis buffer with 20 mM imidazole) and finally eluted with elution buffer (lysis buffer with 200 mM imidazole). The homogeneity of protein was checked on 12% SDS-PAGE gel, and the protein was stored at −80°C for future use. Endotoxin levels checked with limulus amebocyte lysate test (after treatment with polymyxin-B agarose beads) were found to be very low.

### Generation of Rv2004c-Specific Polyclonal Antibody

To raise the polyclonal antibody against recombinant Rv2004c protein, Swiss Albino female mice were maintained in animal house facility at University of Hyderabad. Experiments were performed based on the guidelines of Institutional Ethical Committee. Pre-immune sera were collected from mice before raising polyclonal sera against Rv2004c. 100 µg of recombinant Rv2004c was mixed thoroughly with complete Freund’s adjuvant (Santa Cruz, CA, USA) and injected into each mouse (intraperitoneal). Booster doses were given with incomplete Freund’s adjuvant twice at 2 weeks interval. Antibody titers were measured by ELISA. Mice were sacrificed on the 47th day, and blood was collected and incubated for 4 h at 37°C and overnight at 4°C. Serum was collected from the blood by performing centrifugation and stored at −80°C. Recombinant Rv2004c was detected as 60 kDa single band on western blot when probed with Rv2004c-specific polyclonal sera (Figure S1 in Supplementary Material), but not with pre-immune sera.

### Indirect ELISA with Latent and Active TB Sera Samples

To check for the humoral immune response of LTBI, PTB, and HC against Rv2004c protein, indirect ELISA was carried out as described earlier (25). In brief, approximately 500 ng (per well) recombinant Rv2004c was coated on ELISA plate in bicarbonate buffer and incubated overnight at 4°C. Plate was blocked with 0.2% BSA for 2 h at room temperature and washed with 1× PBST + 0.05% Tween 20 for five times by soaking the plate for 1 min each time. Sera samples were diluted to 1:250 in 1× PBS (containing 0.2% BSA); 100 µl of diluted sera samples were added into each well and incubated for 3 h at room temperature. Plate was washed five times with 1× PBST and incubated with 1:15,000 diluted HRP-conjugated antihuman IgG secondary antibody (Sigma Aldrich, USA) for 1 h. Plate was washed five times with 1× PBST and developed in dark with 3,3′,5,5′-tetramethylbenzidine substrate solution (eBiosciences, USA) for 30 min. The reaction was stopped by adding 2 N sulfuric acid, and readings were measured at 450 nm by correcting the wavelength at 570 nm using ELISA reader (TECAN Infinite M200).

### Rv2004c and TLR-2 Interaction

#### HEK-Blue TLR-2/TLR-4™ Reporter Assay

HEK-Blue hTLR-2/TLR-4™ (InvivoGen, USA) is an engineered cell line co-transfected with TLR-2/TLR-4 and secreted embryonic alkaline phosphatase (SEAP) reporter gene. The SEAP is placed under the control of IFN-β promoter conjoined with five NF-κB and AP-1 binding sites. Interaction of ligand with TLR-2 results in the activation of NF-κB that induces the synthesis of SEAP, which can change the pink color detection medium into purple color; the latter can be measured spectrophotometrically at 620 nm. Approximately 50,000 cells (HEK-Blue hTLR-2) and 25,000 cells (HEK-Blue hTLR-4) were seeded in each well and treated with different concentrations of Rv2004c (10, 50, 100, and 200 ng) for a period of 6 h in presence of HEK-Blue™ detection medium. Recombinant His-tagged Rab5 and FSL-1 (*Mycoplasma salivarium* derived lipoprotein) were used as negative and positive controls, respectively.

#### Immunoprecipitation Assay

To identify the interaction of Rv2004c with TLR-2 surface receptors, whole-cell protein was isolated from THP-1 cells using whole-cell extraction buffer (WEB) (200 mM Tris, 2 mM EDTA, 0.1% NP40, 250 mM NaCl) containing protease inhibitor cocktail. 50 µl of Dynabeads^®^ (Life Technologies, USA) was washed twice with WEB and incubated at room temperature with anti-Rv2004c polyclonal antibody for 2 h followed by incubation with 10 µg of recombinant Rv2004c or recombinant Rab5 (negative control) for 4 h. Later, the Dynabeads, antibody and protein complex was washed twice with WEB, and the complex was incubated with whole-cell lysate overnight at 4°C. The formed complex was again washed twice with WEB and resuspended in 30 µl 4× SDS buffer and heated at 98°C for 10 min. The samples were subjected to SDS electrophoresis, and the bands were transferred on to PVDF membrane. The blot was probed with antihuman-TLR-2 (Novus Biologicals, USA) and anti-His antibody (Sigma Aldrich, USA) for TLR-2 and recombinant Rv2004c, respectively, followed by protein A HRP conjugate (Abcam, Cambridge, UK). The blot was then developed in versa doc (Bio-rad) using Super signal west Pico Kit (Thermo Fischer Scientific, USA).

### Analysis of Surface Expression of TLR-2 by qRT-PCR and Flow Cytometry

1 × 10^6^ differentiated THP-1 cells were treated with different concentrations of Rv2004c (10 and 100 ng) for a period of 24 h. Trichostatin A (TSA) (250 ng) treated THP-1 cells served as positive control. Total RNA was isolated using Trizol method. 2 µg of DNase treated RNA was converted into cDNA by using first strand cDNA synthesis kit (Invitrogen, Life Technologies, USA). Quantitative real-time PCR was performed in Eppendorf real-time PCR machine by utilizing primers listed in Table S1 in Supplementary Material. 10 µl reaction mixture included 5 µl SYBR green (Takara, Japan), 0.2 µl of each primer, and 40 ng of cDNA and DNase free water. The reaction was incubated at 95°C (10 min), 95°C (15 s), 58°C (15 s), and 72°C for 15 s. Calculation was done by using ΔΔCT method. Glyceraldehyde-3-phosphate dehydrogenase was used as housekeeping gene for normalization of transcript levels. For flow cytometry analysis, THP-1 cells were harvested, rinsed using 1× PBS and incubated with goat antihuman TLR-2 antibody (R&D Systems, USA) or isotype matched goat IgG2a for 1 h at 4°C. Cells were rinsed once again with 1× PBS + 2% FBS and probed with donkey anti-goat IgG FITC-conjugated secondary antibody (Santa Cruz, CA, USA). Unstained cells were used as negative control. The intensity of fluorescence was measured by BD FACS Canto II flow cytometer (BD Biosciences, USA). Data were analyzed by using FlowJo software (Tree Star Inc., USA).

### Cytokine Assays

Human PBMCs were isolated using Ficoll-Histopaque (Sigma, USA) gradient method. 1 × 10^6^ PBMCs were seeded into each well by suspending in RPMI1640 complete media containing 10% FBS. Cells were treated with different concentrations of Rv2004c (10, 100, and 1,000 ng/ml) or proteinase K (Pro K)-treated Rv2004c (negative control) or LPS (100 ng/ml; positive control), and supernatant was collected at 24 h time point and stored at −80°C. A total of around 0.2 × 10^6^ THP-1 cells (per well) were seeded into 24-well plate and differentiated with phorbol 12-myristate 13-acetate (Sigma, MO, USA) for 48 h. Cells were treated with different concentrations of recombinant Rv2004c as mentioned for PBMCs. As negative controls, untreated cells and cells treated with Pro K digested Rv2004c were used. THP-1 cells that were preincubated with anti-TLR-2 antibody (3 µg/ml) before Rv2004c (1 µg/ml) induction were also used as one of the controls. Supernatants were collected after 24 and 48 h time intervals. Analysis of pro-inflammatory cytokines such as TNF-α, IL-8, IL-1β, IFN-γ, and IL-12 cytokines was carried out using sandwich ELISA kit (eBiosciences, USA) according to the manufacturer’s instructions.

### Western Blot Analysis

7 × 10^6^ differentiated THP-1 cells were treated with 1 µg of Rv2004c for 24 h. Untreated cells and cells treated with LPS (100 ng/ml) served as negative and positive controls, respectively. Whole-cell protein from THP-1 cells was prepared according to the protocol described earlier (26). 100 µg of total protein was subjected to SDS PAGE and transferred onto PVDF membrane. The blots were probed with mouse antihuman p65 NF-κB antibody (Abcam, USA) followed by goat anti-mouse secondary antibody (Sigma Aldrich, USA). Blots were developed in versa doc (Bio-rad) using Super signal west Pico Kit (Thermo Fischer Scientific, USA).

### Statistical Analysis

GraphPad prism 5.01 software was used to perform statistical tests. Wherever required, Student’s *t*-test, Mann–Whitney’s *U* test or the Wilcoxon rank sum test, and one-way ANOVA were performed for statistical analyses of the results. *p* values < 0.05 were considered as statistically significant.

## Results

### Rv2004c is Specifically Recognized in LTBI Compared to that of PTB and HC Cohorts

**Figure 1:**
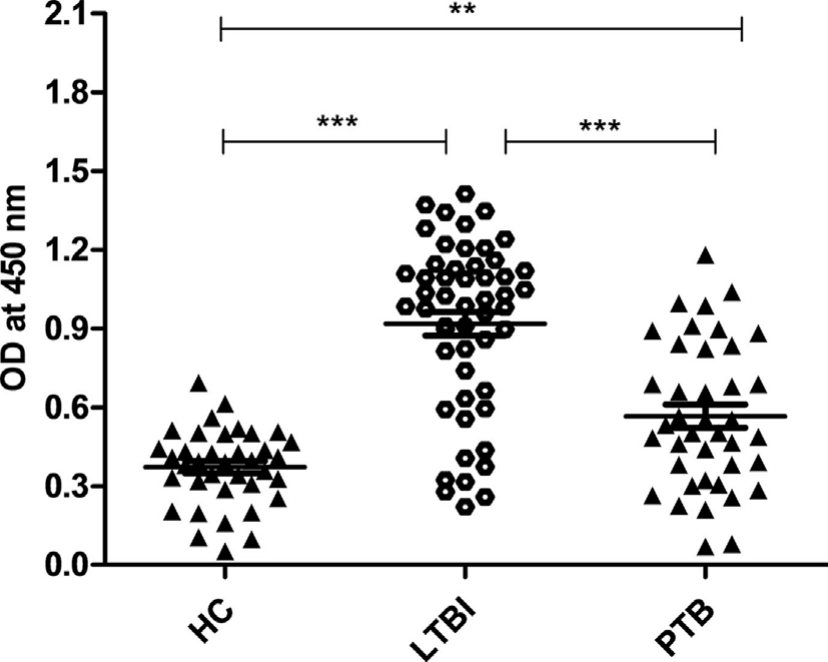
Humoral immune response of Rv2004c in healthy control (HC), latent tuberculosis infection (LTBI), and pulmonary tuberculosis (PTB) groups was performed by indirect ELISA method. Immunoreactivity to Rv2004c was significantly higher in sera samples of LTBI compared to that of PTB and HC cohorts. The median values of each population were represented with a horizontal line including ±SD. Data represent three independent experiments. Statistical analysis was carried out by using Mann–Whitney *U* test. *p* values were represented with asterisk symbol (**p < 0.01, ***p < 0.001).

Latently infected population was screened using QuantiFERON-TB Gold (QFT^®^) kit. Out of 89 samples screened, 52 were latent positive, and 37 were negative for this test (we considered these 37 as HCs). Since Rv2004c is a cell surface protein, we expected that it could induce strong B-cell response in LTBI population. We evaluated IgG antibody response against Rv2004c protein in latently infected (*n* = 52) and active TB groups (*n* = 40) by comparing with HCs (*n* = 37). Our data demonstrated that antibody titers for Rv2004c were significantly higher in LTBI group followed by PTB cases, as compared to HC (*p* < 0.0001). Higher immunoreactivity of LTBI group than that of PTB cases might be due to higher expression of Rv2004c during latency. The median values of HC, LTBI, and PTB were 0.3, 0.9, and 0.6, respectively (Figure 1).

### Rv2004c Interacts with TLR-2 Surface Receptor

**Figure 2:**
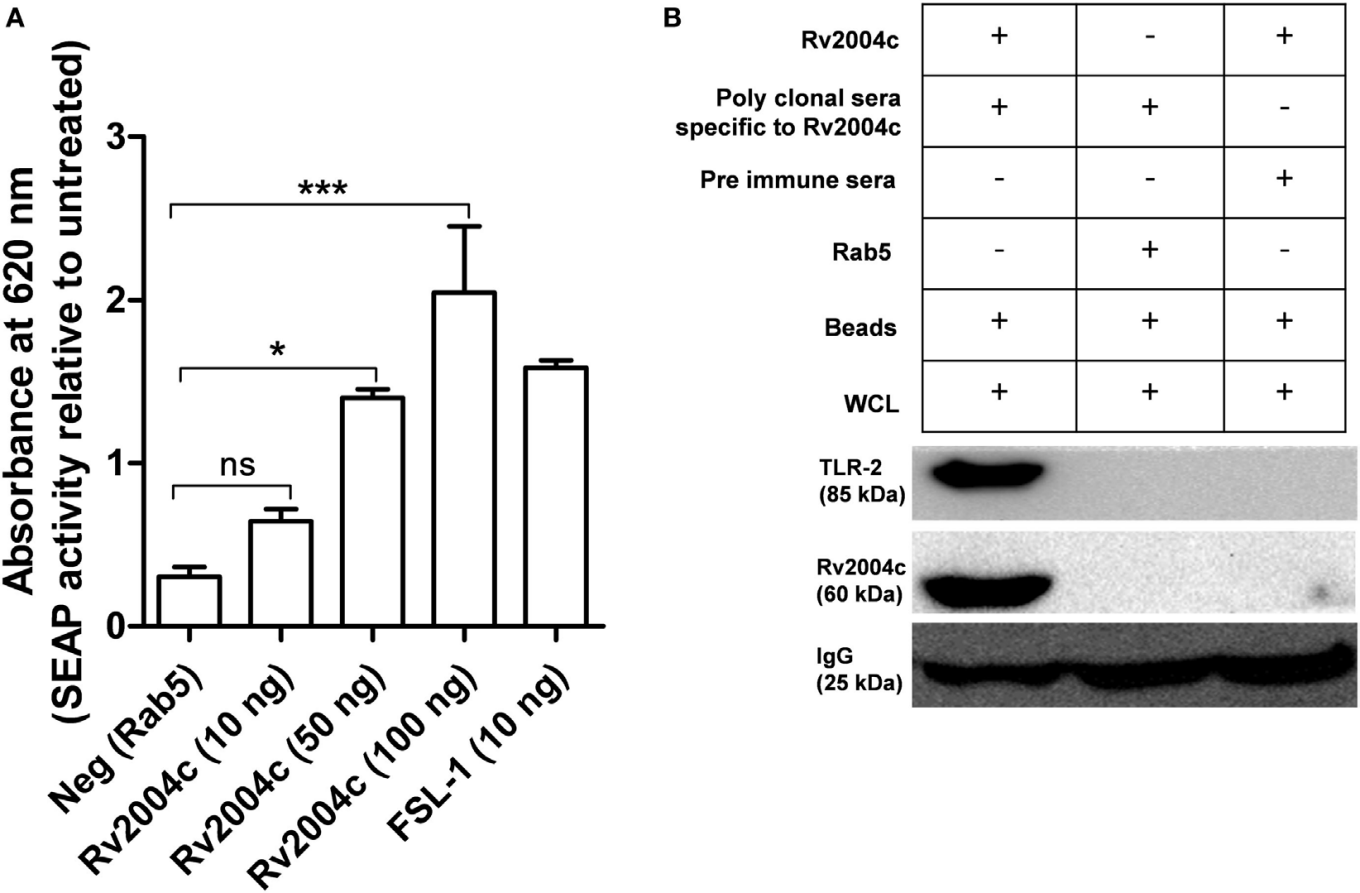
Interaction of Rv2004c with toll-like receptor (TLR)-2. **(A)** HEK-Blue hTLR-2™ reporter assay: cells were treated with different concentrations of Rv2004c (10, 50, and 100 ng) for 6 h, and the color formation was measured at 620 nm in ELISA reader. Recombinant Rab5 was used as a negative control. Cells treated with 10 ng of *Mycoplasma salivarum*-derived lipoprotein (FSL-1) served as positive control. Data were represented as mean ± SD of three independent experiments. One-way ANOVA followed by Tukey’s multiple comparison test was performed for statistical analysis. *p* values were represented with an asterisk symbol. (ns = non significant, *p < 0.05, ***p < 0.001). **(B)** Immunoprecipitation for binding analysis of TLR-2 with Rv2004c: the THP-1 cell lysates incubated with Rv2004c (100 µg) were immunoprecipitated using anti-Rv2004c polyclonal antibody conjugated to magnetic beads. The formed immune complex was probed with anti-TLR-2 and anti-His antibodies to detect TLR-2 and Rv2004c. Cell lysate incubated with pre-immune sera + Rv2004c and cell lysate incubated with anti-Rv2004c polyclonal antibody + Rab5 (another His-tagged recombinant protein) were used as negative controls.

Previous studies demonstrated that most of the Mtb ligands interact *via* TLR-2 or TLR-4 (19, 20, 27). Hence, we were interested to see the interaction between Rv2004c and TLR-2/TLR-4, which triggers the downstream signaling. Host receptors involved in the interaction with Rv2004c were identified by HEK-Blue hTLR-2™ reporter assay. Rv2004c induced SEAP secretion from HEK-Blue hTLR-2 cells in a dose-dependent manner. Positive control (FSL-1) also stimulated HEK-Blue hTLR-2™ cells to secrete SEAP. Recombinant Rab5 (negative control) did not induce SEAP activity (Figure 2A). HEK-Blue hTLR-4™ cells treated with Rv2004c failed to augment SEAP activity (Figure S2 in Supplementary Material). This indicated that Rv2004c interaction is TLR-2 specific. Moreover, Rv2004c interaction with TLR-2 was further reinforced by immunoprecipitation assay. Anti-Rv2004c polyclonal sera captured Rv2004c + TLR-2 complex, which was visualized on western blot upon probing with anti-TLR-2 antibody and anti-His antibody. Anti-Rv2004c poly clonal sera incubated with recombinant Rab5 and whole-cell lysate did not capture TLR-2. This ruled out the non-specific interaction of poly-clonal sera or any other bacterial components eluted during recombinant protein preparation with TLR-2 (Figure 2B).

### Rv2004c Upregulates TLR-2 Expression

**Figure 3:**
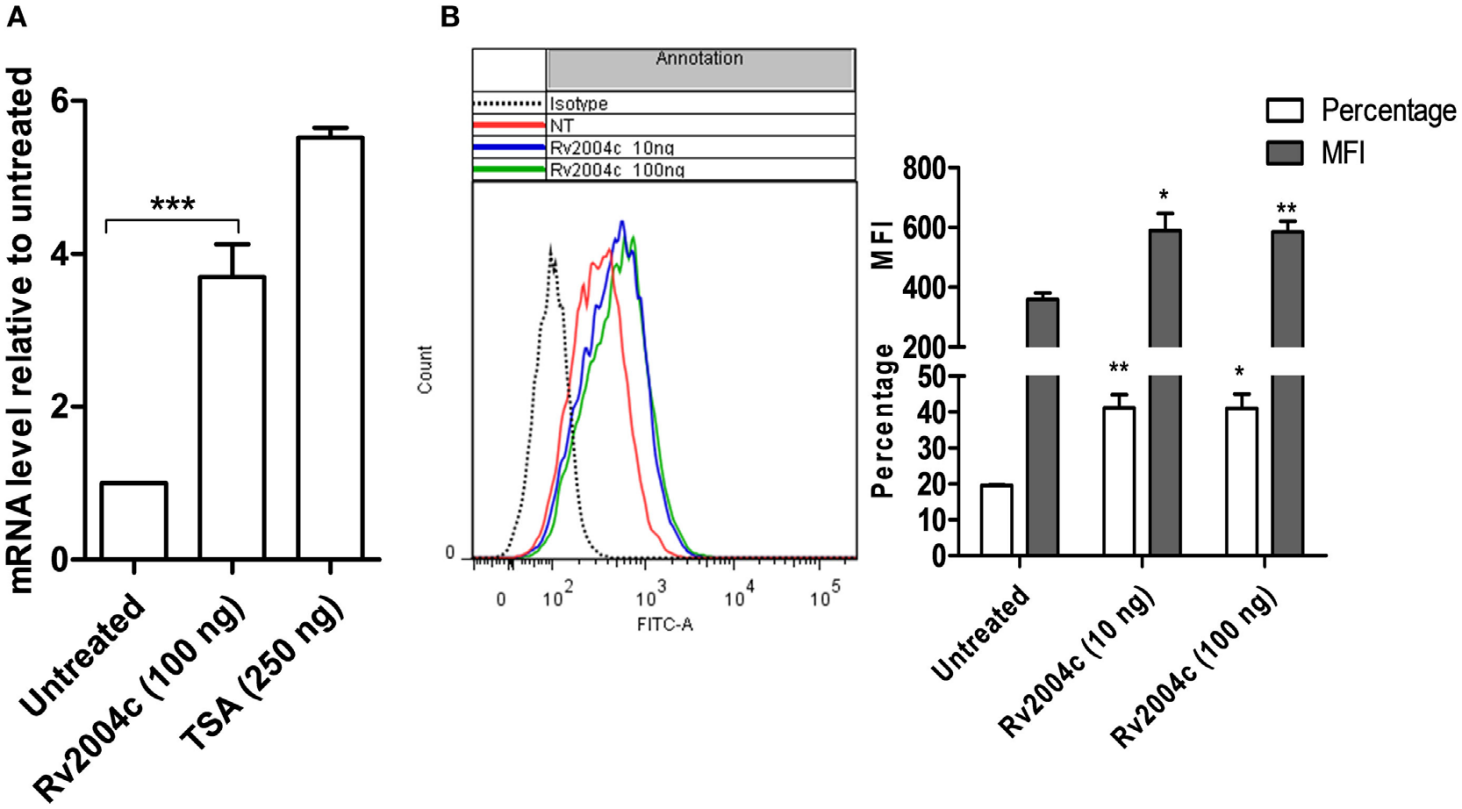
Upregulation of toll-like receptor (TLR)-2 expression upon treatment with Rv2004c. **(A)** Fold increase in the TLR-2 expression at mRNA level was quantified in differentiated THP-1 cells that were treated with 100 ng of Rv2004c and 250 ng of Trichostatin A (TSA) (positive control) by real-time PCR. Untreated cells served as negative control. **(B)** Surface expression of TLR-2 on differentiated THP-1 cells was analyzed by flow cytometry upon treatment with 10 and 100 ng of Rv2004c for 24 h. Untreated cells and cells probed with isotype-matched control were used as negative controls. Data from three experimental replicates (mean ± SD) were represented as a shift in histogram peaks (left side), percentage of TLR-2-expressing cells and mean fluorescence intensity (MFI) (right side). One-way ANOVA followed by Tukey’s multiple comparison test was performed for statistical analysis. In all the graphs, *p* values were represented with an asterisk symbol (*p < 0.05, **p < 0.01, ***p < 0.001).

Besides binding to TLR-2, Rv2004c also enhanced the expression of TLR-2, which was analyzed by quantitative real-time PCR and flow cytometry. THP-1 cells that were treated with Rv2004c and TSA (positive control) showed increased expression of TLR-2 mRNA when compared with untreated cells (Figure 3A). Furthermore, upregulation of TLR-2 was confirmed at protein level by flow cytometric analysis in Rv2004c-treated THP-1 cells (Figure 3B).

### Rv2004c Induces Pro-inflammatory Response

**Figure 4:**
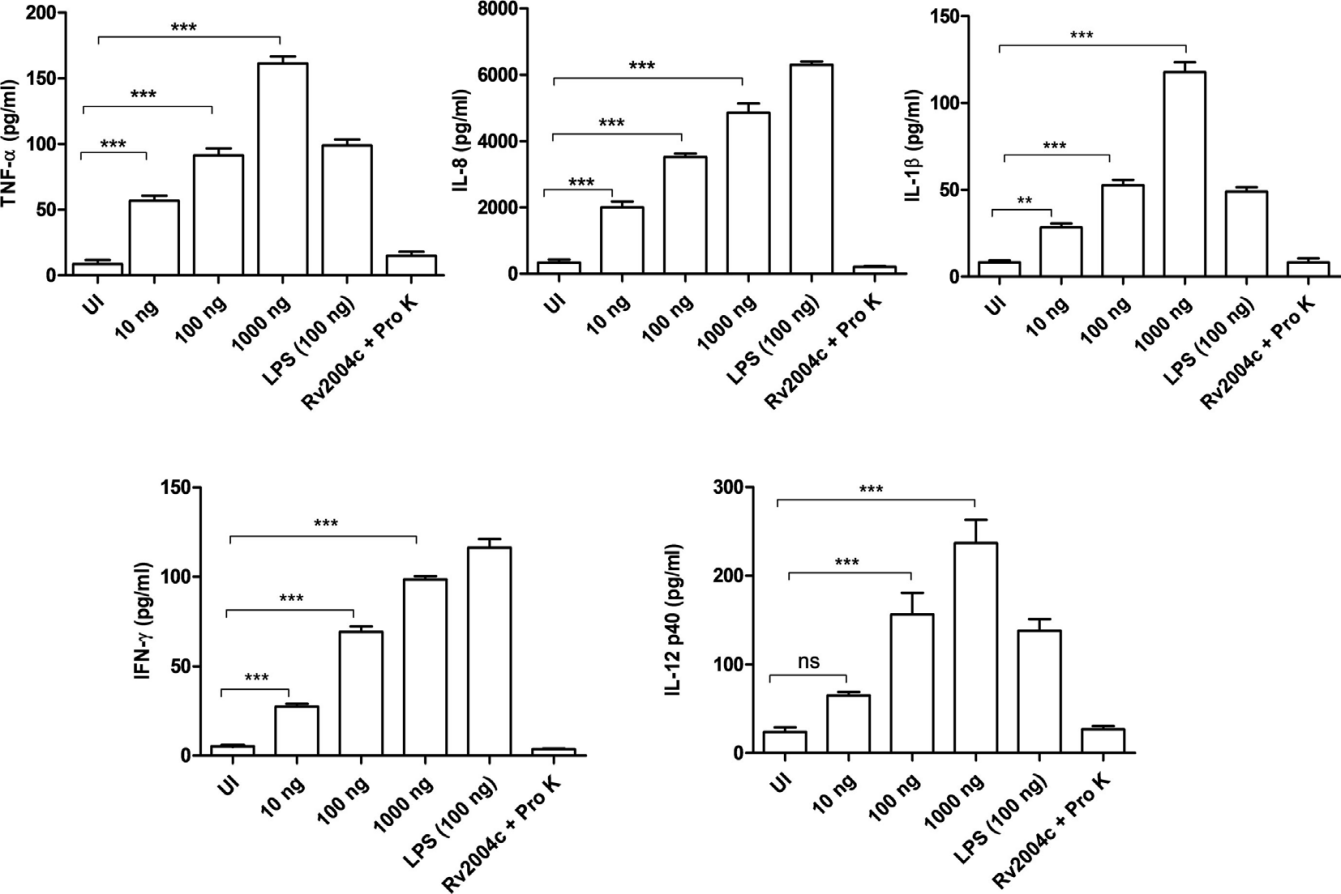
Pro-inflammatory cytokine response of peripheral blood mononuclear cells (PBMCs) stimulated with Rv2004c. Sandwich ELISA was performed from the supernatants of PBMCs treated with different concentrations of Rv2004c for 24 h. We detected dose-dependent increase in TNF-α, IL-8, IL-β, IFN-γ, and IL-12 p40. Un-induced (UI) cells and cells treated with proteinase K (Pro K) digested Rv2004c (Rv2004c + Pro K) served as negative controls. PBMCs stimulated with LPS (100 ng/ml) served as positive control. Data represent mean ± SD of three individual experiments. One-way ANOVA followed by Tukey’s multiple comparison test was performed for statistical analysis. *p* values were represented with asterisk symbols (ns = non significant, **p < 0.01, ***p < 0.001).

**Figure 5:**
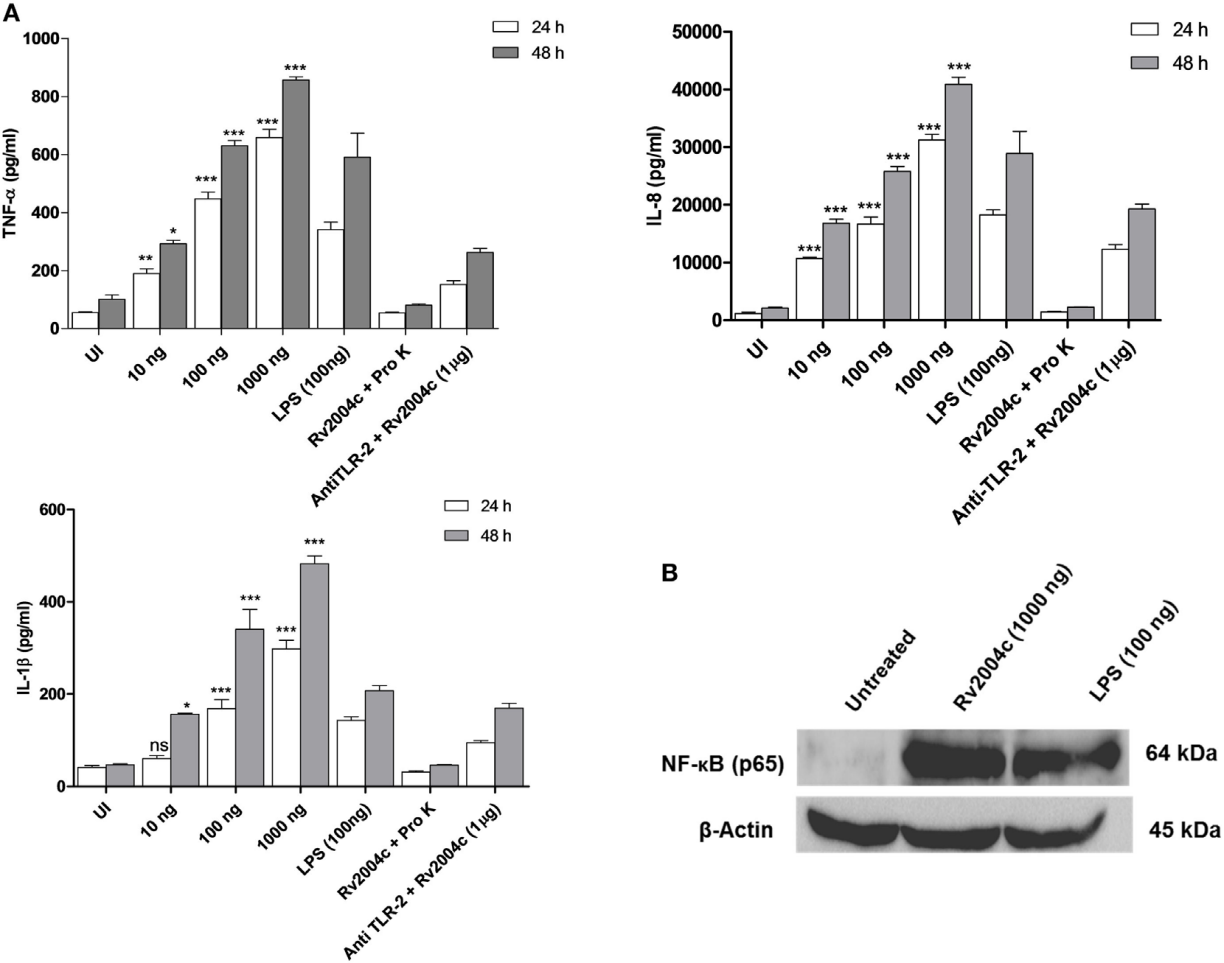
Pro-inflammatory cytokine response of THP-1 cells treated with Rv2004c. **(A)** Differentiated THP-1 cells treated with different concentrations of Rv2004c secreted TNF-α, IL-8, and IL-1β in a dose- and time-dependent manner. Positive and negative controls for THP-1 cytokine assay were same as mentioned for peripheral blood mononuclear cells. To check whether the cytokine response triggered by Rv2004c was through toll-like receptor (TLR)-2 interaction, Rv2004c stimulated THP-1 cells that were preincubated with anti TLR-2 antibody were assessed for TNF-α, IL-8, and IL-1β cytokines response. Data represent mean ± SD of three individual experiments. One-way ANOVA followed by Tukey’s multiple comparison test was performed for statistical analysis. *p* values were represented with asterisk symbols (*p < 0.05, **p < 0.01, ***p < 0.001). **(B)** Analysis of NF-κB activation in Rv2004c treated THP-1 cells. Western blot with antibodies specific to phosphorylated p65 was performed to detect active form of NF-κB. β-Actin was used as an internal control, and LPS-treated cells were used as a positive control.

Human PBMCs treated with Rv2004c at a concentration of 10 ng to 1 µg secreted TNF-α, IL-8, IL-1β, IFN-γ, and IL-12 in a dose-dependent manner (Figure 4). Similarly, THP-1 cells treated with Rv2004c showed dose- and time-dependent cytokine responses (TNF-α, IL-8, and IL-1β) (Figure 5A). Cells treated with LPS (100 ng/ml) functioned as positive control and induced strong cytokine response whereas negative controls (unstimulated cells and cells that were treated with Pro K digested Rv2004c) did not show such an effect. Rv2004c-treated THP-1 cells that were preincubated with anti TLR-2 neutralizing antibody secreted less TNF-α, IL-8, and IL-1β. Rv2004c-treated THP-1 cells produced activated form of NF-κB (p65), which was visualized by probing the whole-cell lysate with antiphospho NF-κB antibody (Novus Biological, USA) whereas in unstimulated THP-1 cells active form of NF-κB was not detected (Figure 5B). This indicates that Rv2004c activates the NF-κB signaling pathway in response to its interaction with TLR-2 and induces the pro-inflammatory cytokine response.

## Discussion

*Mycobacterium tuberculosis* is the etiological agent of TB and still remains a major global health burden. It infected about 10.4 million people, newly, and killed about 1.8 million in 2015, globally (28). Next to HIV infection, TB is the leading cause of death due to a single bacteriological agent (29). Mtb can cause primary active infection, or it can enter into dormant state and persist for years, asymptomatically, in LTBI individuals (30). Resuscitation of dormant bacilli is one of the major causes of active TB. Latent-specific antigens that strongly induce immune responses are likely to serve as potent serodiagnostic markers/vaccine candidates that can help control LTBI. In this study, we evaluated the serodiagnostic potential of Rv2004c in LTBI. Our results showed that Rv2004c was significantly recognized in LTBI individuals compared to that of PTB and HC cohorts (Figure 1). The cohort collected from Hyderabad region included people from various regions of Telangana and Andhra Pradesh. This is the first report from India showing humoral immune response in LTBI. Validating the humoral immune response of Rv2004c in different ethnic populations and other high burden settings is warranted. The prognostic value of a novel LTBI diagnostic tool can be further enhanced through a combined approach by screening and involving other putative DosR regulon antigens in addition to Rv2004c. Serological potential of other latent-specific antigens was also reported (31). Hence, latent-specific DosR regulon antigen Rv2004c might be a potential target for inducing antibody-mediated immunity in current TB (BCG) vaccine strategies to prevent LTBI.

Besides its serodiagnostic potential, we identified the interacting surface receptors of Rv2004c. Previously, it was reported that peptide fragments of Rv2004c interact with the surface of U-937 macrophages and A-547 epithelial cells (21). This stimulated our interest to further explore the interacting surface receptors of Rv2004c. We also demonstrated the interaction of recombinant Rv2004c with TLR-2 by HEK-Blue™ reporter assay. This interaction was further confirmed by immunoprecipitation assay (Figures 2A,B). Moreover, Rv2004c enhanced the TLR-2 expression in THP-1 cells, which was confirmed by qRT-PCR and flow cytometric analyses (Figures 3A,B). It is an acceptable practice to study the functional properties of hypothetical proteins by expressing them in prokaryotic expression system as it is not possible to overexpress and purify these proteins in mycobacterial systems. Thus, recombinant Rv2004c obtained from *E. coli* expression system demonstrated the possible interaction with TLR-2 leading to plausible downstream signaling events during *M. tuberculosis* infection.

During Mtb infection, the first immune cells that encounter the bacilli in the lungs are the alveolar macrophages. Recognition of different virulence factor of Mtb by macrophages is critical for the development of adaptive immune response. Interaction of different cell wall components such as lipoprotein and glycolipids of Mtb occurs through TLR-2 indicating that these innate immune receptors play a vital role in defense against mycobacterial infections (32). It has been reported that Mtb lipoproteins LpqH (19 kDa protein), LprA (Rv1270), and LprG (Rv1411c) were recognized by TLR-2 receptors (33). Previous studies demonstrated that lipopeptides of Mtb involve in TLR-2-dependent maturation of dendritic cells and activation of naïve T-cells resulting in initiation of adaptive immune responses (34). Besides lipoproteins, glycolipids such as phosphatidylinositol mannoside, lipoarabinomannan, and lipomannans are also recognized by TLR-2 (33). Role of TLR-2 in Mtb antigen recognition during pathogenesis and susceptibility of host to Mtb infection in TLR-2-deficient mice was well established (16, 35). TLR-2-deficient mice have shown reduced bacterial clearance and failed to form functional mycobactericidal granulomas (32). Modulation of TLR-2 expression by Mtb antigens has been reported (36). In our earlier studies, other DosR genes like DATIN and Rv3131 were shown to interact with TLR-2 and Rv3131 upregulated TLR-2 expression (26, 37).

Interaction of Mtb antigens with TLR-2 usually triggers a downstream signaling pathway resulting in the secretion of pro- or anti-inflammatory cytokines that are critical for recruitment of macrophages, other immune cells, and maintenance of granulomatous structure. Mtb relentlessly maintains the balance between pro- and anti-inflammatory repertoire by manipulating the host innate immune system in local milieu of granuloma for its existence (37, 38). Role of TNF-α in the formation and maintenance of granuloma has been extensively reported (39, 40). TNF-α is essential for activation of phagocytes and control of chronic infection with Mtb. TNF-deficient mice showed exacerbated immune responses and granuloma disintegration (41). Previously, it was reported that Mtb induces strong IL-1 β cytokine secretion, which involves in-host resistance to mycobacterial growth (42). IL-12 and IFN-γ are critical cytokines that initiate the acquired immune responses during Mtb infection. Humans deficient with IL-12 and IFN-γ pathways have been associated with susceptibility to TB (39). IL-12 initiates the T-helper 1 cells to secrete IFN- γ, which is essential for control of bacterial burden in granulomas (43). On the other hand, IL-10, an anti-inflammatory cytokine modulates macrophage functions and suppresses their ability to secrete TNF-α and IL-12 and controls pro-inflammatory responses (39). Ultimately, pulmonary inflammation maintains dynamic equilibrium in granuloma and prevents the dissemination of infection to new sites. In the current study, Rv2004c induced strong pro-inflammatory cytokine responses (TNF-α, IL-8, and IL-1β) in THP-1 cells in a dose- and time-dependent manner whereas PBMCs showed only a dose-dependent increase of TNF-α, IL-8, IL-1β, IFN-γ, and IL-12 (Figure 4). In another study, Rv2004c was shown to stimulate strong IFN-γ response in Japanese LTBI population (24). In an earlier report, other DosR regulon gene, Rv2626c stimulated TNF-α and IL-12 in RAW macrophages by activating NF-κB downstream signaling pathway and induced strong IFN-γ response in active TB patients (44). In this study, we also demonstrated phosphorylation of NF-κB in Rv2004c treated macrophages by western blot analysis (Figure 5B). Given this, it might be possible that interaction of Rv2004c with TLR-2 activates the NF-κB pathway, leading to secretion of pro-inflammatory cytokines.

In conclusion, the Mtb DosR antigen, Rv2004c might possibly interact with TLR-2 inducing cytokine response to enable the endurance of Mtb in the granulomas during latent infection. On the other hand, Rv2004c elicited strong humoral immune responses in LTBI individuals compared to PTB and HC cohorts. Our results suggest a possible use of Rv2004c as a putative serodiagnostic marker for latent TB or an attractive target for vaccine development as it can induce both humoral as well as cellular immune responses. However, identification of the molecular functions of Rv2004c, its requirement during latent infection, its role in disease reactivation and the nature and extent of the T-cell responses it triggers in LTBI individuals will be some of the interesting issues to be addressed in future studies.

## Ethics Statement

For experiments involving human samples, World Medical Association (Declaration of Helsinki) ethics code was followed. Written informed consent was obtained from all the individuals, and the study was approved by both Institutional Ethics and Biosafety Committees of the University of Hyderabad and Bhagwan Mahavir Hospital and Research Centre.

## Author Contributions

SD carried out experiments, analyzed the data, and wrote the manuscript. VP helped in execution of the experiments and analyzing data. NA conceptualized the study, provided funding, critically reviewed the manuscript, and extended overarching supervision throughout the study.

## Conflict of Interest Statement

The authors declare that the research was conducted in the absence of any commercial or financial relationships that could be construed as a potential conflict of interest. The reviewer, TD, and handling editor declared their shared affiliation, and the handling editor states that the process nevertheless met the standards of a fair and objective review.
