# Supplementary material for: Mycobacterium tuberculosis DosR Regulon Gene Rv2004c Encodes a Novel Antigen with Pro-inflammatory Functions and Potential Diagnostic Application for Detection of Latent Tuberculosis
Source: Front Immunol. 2017 Jun 24;8:712. doi: 10.3389/fimmu.2017.00712 (PMC5483032; doi:10.3389/fimmu.2017.00712)
Supplement: Supplementary file 1 [file Data_Sheet_1.pdf]

**Supplementary Material**

Doddam SN, Peddireddy V and Ahmed N (2017) Mycobacterium tuberculosis DosR Regulon Gene Rv2004c Encodes a Novel Antigen with Pro-inflammatory Functions and Potential Diagnostic Application for Detection of Latent Tuberculosis. Front. Immunol. 8:712. doi: 10.3389/fimmu.2017.00712

**Table S1:** List of primers used in this study. Restriction sites were highlighted with bold letters

| S. No | Primer name | Sequence | Reference |
|---|---|---|---|
| 1 | Rv2004c | F-5’CG**GGATCC**ATGGACTCACCGACCAACGACG3’ | This study |
| | | R-5’CC**AAGCTT**TCA GAT CGC GCT GCG CCA GATGTG3’ | |
| 2 | qRT_TLR2 | F-5’GGCCAGCAAATTACCTGTGTG 3’ | (1) |
| | | R-5’AGGCGGACATCCTGAACCT 3’ | |
| 3 | qRT_GAPDH | F-5’GGAAGGTGAAGGTCGGAGTC3’ | (2) |
| | | R-5’TGAGGTCAATGAAGGGGTCA3’ | |

M 1 2 3

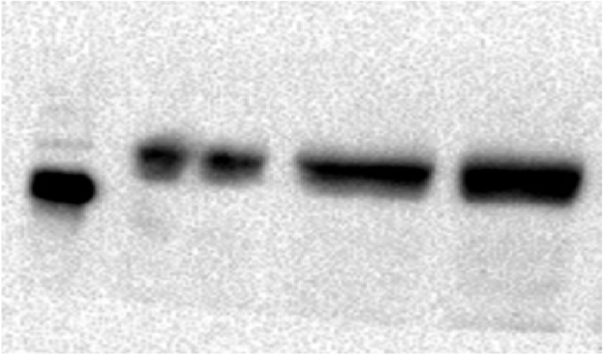

**Rv2004c (60 kDa)**

**Figure S1: Specificity of mouse polyclonal sera raised against Rv2004c.** Polyclonal antibody was probed against Rv2004c at 1:2000 dilution in PBST buffer for 3 h at room temperature. Lanes 1- 3 represent 10, 20, 30 µg of Rv2004c, respectively, and M represents marker.

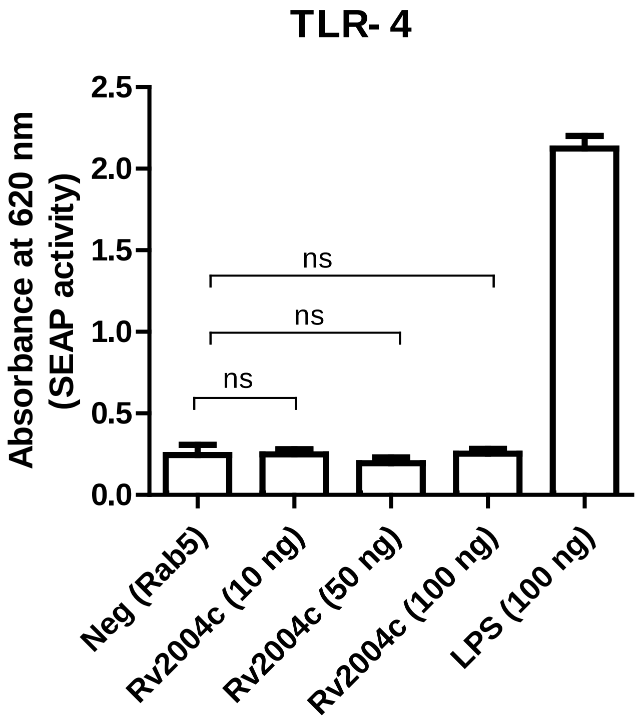


**Figure S2: Analysis of Rv2004c interaction with TLR-4.** Interaction of Rv2004c with TLR-4 was analysed by a cell based colorimetric assay using HEK-blue™ hTLR-4 engineered cell line. To measure the interaction of Rv2004c with TLR-4, HEK-blue™ hTLR-4 cells (25000 per well) were treated with different concentrations of Rv2004c and incubated in the presence of HEK- blue™ detection medium for 7 h at 37 °C in an incubator with 5% $CO_2$. Colour change from pink to purple was monitored using spectrophotometer at 620 nm. LPS was used as positive control and recombinant Rab5 was used as negative control. Data was represented as mean ± SD of three independent experiments. One way ANOVA followed by Tukey's multiple comparison test was performed for statistical analysis (ns = non-significant).

1. **Hayashi F**, **Means TK**, **Luster AD**. 2003. Toll-like receptors stimulate human neutrophil function. Blood **102**:2660–2669.

2. **Moore X-L**, **Lu J**, **Sun L**, **Zhu C-J**, **Tan P**, **Wong M-C**. 2004. Endothelial progenitor cells' "homing" specificity to brain tumors. Gene Ther **11**:811–8.
